# Alcohol and Opioids Modulate Excitatory Inputs to the SCN

**DOI:** 10.1111/acer.70447

**Published:** 2026-11-10

**Authors:** Valerie Vierkant, William Purvines, Ziyi Li, Ling Qi, Peyton Westbo, Xuehua Wang, Jeff R. Jones, David Earnest, Jun Wang

**Affiliations:** ^1^ Department of Neuroscience and Experimental Therapeutics, College of Medicine Texas A&M University Health Science Center Bryan Texas USA; ^2^ Texas A&M Institute for Neuroscience Texas A&M University College Station Texas USA; ^3^ Department of Biology Texas A&M University College Station Texas USA; ^4^ Center for Biological Clocks Research Texas A&M University College Station Texas USA

**Keywords:** alcohol, circadian rhythms, glutamatergic transmission, mu‐opioid receptor, opioids, suprachiasmatic nucleus

## Abstract

**Background:**

Disturbance of circadian rhythms is a hallmark of substance use disorders, with depressant drugs often causing soporific effects such as reduced sleep latency. The suprachiasmatic nucleus (SCN) of the hypothalamus is the central circadian pacemaker in mammals, regulating daily rhythms in physiology and behavior. However, the cellular mechanisms through which depressants alter SCN function remain poorly defined.

**Methods:**

We used whole‐cell patch clamp electrophysiology in coronal brain slices to examine how alcohol and opioids modulate excitatory glutamatergic transmission onto SCN neurons. Ethanol effects were examined acutely and following chronic exposure paradigms. Optogenetic stimulation was used to activate either retinohypothalamic tract (RHT) input or μ‐opioid receptor‐expressing (MOR^+^) terminals, and MOR agonists were used to assess opioid‐mediated effects on synaptic transmission.

**Results:**

We show that acute application of ethanol enhances SCN firing rates during the inactive locomotor stage of circadian rhythms (daytime for rodents). In contrast, chronic alcohol exposure reduces glutamatergic drive. We also found that activating MOR^+^ terminals via an excitatory channelrhodopsin transgenically expressed in MOR+ neurons produced bidirectional modulation of SCN firing and that MOR+ inputs formed functional glutamatergic synapses onto SCN neurons. We also found that chronic alcohol consumption significantly altered MOR‐dependent modulation of SCN neuronal activity, demonstrating an interaction between ethanol exposure and opioidergic signaling. Notably, MOR‐mediated transmission could be suppressed by the MOR agonists DAMGO and fentanyl.

**Conclusions:**

Together, these findings reveal that both alcohol and opioids modulate glutamatergic input to the SCN. This work establishes the SCN as a novel target of depressant substances and highlights glutamatergic transmission as a key point of vulnerability in circadian dysregulation associated with substance use.

## Graphical abstract

This study investigated how depressant drugs modulate the “central circadian clock,” the suprachiasmatic nucleus (SCN), through patch‐clamp electrophysiology. Acute alcohol bath application during the inactive circadian phase increased SCN spontaneous firing. Chronic alcohol consumption decreased the amplitude of oEPSCs when VGlut2‐Cre;Ai32 afferents were stimulated with channelrhodopsin. Chronic alcohol consumption altered SCN response to MOR‐Cre;Ai32 stimulation, biasing it toward excitatory responses. This work establishes the SCN as a novel target of depressant substances and highlights glutamatergic transmission as a key point of vulnerability in circadian dysregulation associated with substance use.

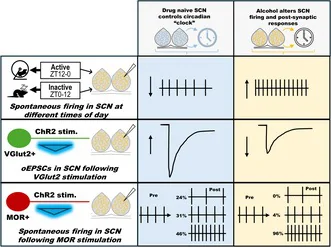

## 1 Introduction

Aberrant substance use is a global public health crisis, with 16.8% of the US population having a diagnosable substance use disorder (SUD) (Administration 2025). Among drugs of abuse, central nervous system depressants such as alcohol and opioids are heavily used and often lead to effects such as compulsive drug seeking, loss of intake control, and cognitive impairment (Koob and Volkow 2010; Ma et al. 2022; Vierkant et al. 2024; Xie, Huang, et al. 2026). Beyond these immediate impairments, the sedative nature of alcohol and opioids can alter natural sleep‐related rhythms, leading to circadian dysregulation that exacerbates drug‐related pathology (Koob and Colrain 2020; Meyrel et al. 2020). In particular, alcohol use has been shown to alter circadian phase and phase‐shifting (Seggio et al. 2009; the ability of an organism to maintain biological rhythms and for them to adjust relative to external cues), sleep latency (Ebrahim et al. 2013; Feige et al. 2006; the length of time it takes for an organism to fall asleep), and melatonin secretion (Kurhaluk 2021). Similarly, opioid use, including prescription analgesics and illicit drugs such as fentanyl, is also associated with disrupted circadian timing (Mehranfard et al. 2025) and increased prevalence of sleep disorders (Bumgarner et al. 2023). However, despite the fact that drug use has been clearly associated with circadian dysregulation (Hasler et al. 2012; Kalsbeek et al. 2006), the cellular and synaptic mechanisms by which these depressants alter circadian timing remain incompletely understood.

Circadian rhythms regulate a wide array of physiological processes, including sleep–wake cycles, hormone release, metabolism, and reward‐seeking behavior (Depoy et al. 2017; Jones, Chaturvedi, et al. 2021). These rhythms are generated and maintained by the suprachiasmatic nucleus (SCN), a bilateral structure in the ventral hypothalamus that functions as the central circadian pacemaker in mammals (Bell‐Pedersen et al. 2005; Jones et al. 2015). The SCN receives dense glutamatergic innervation from the retinohypothalamic tract (RHT), originating from intrinsically photosensitive retinal ganglion cells (ipRGCs), which convey photic input to entrain the circadian clock to the external light–dark cycle (Fernandez et al. 2016; Starnes and Jones 2023). Within the SCN, coordinated synaptic and intrinsic properties translate photic input into rhythmic output, organizing the timing of downstream physiological and behavioral functions (Jones, Tackenberg, and Mcmahon 2021).

One point of convergence for alcohol‐ and opioid‐related disturbances may be their shared influence on glutamatergic signaling, an aspect critical for SCN function. Glutamate is well known to regulate phase resetting in the SCN, with glutamatergic bath application causing phase shifts (Paul Franken et al. 1999; Tetsuo Shirakawa 1994). Alcohol has been reported to modulate excitability and synaptic transmission in multiple brain regions, including both enhancement and suppression of excitatory drive depending on context and region (Harrison et al. 2017; Ma et al. 2022; Xie et al. 2023). Interestingly, alcohol has previously been shown to inhibit glutamate‐induced phase shifting of the SCN clock (Prosser and Glass 2015), further indicating glutamatergic transmission is involved in mediating alcohol's effects on circadian rhythms. Specifically, multiple ex vivo investigations have shown that alcohol application consistently inhibits the effects of exogenous glutamate on SCN rhythms and phase shifting (Prosser et al. 2008; Prosser and Glass 2009). These reports are corroborated by findings that acute exposure to alcohol in vivo has the same effect on photic shifts in free‐running conditions (Brager et al. 2011). Beyond acute alcohol exposure, ex vivo examinations show decreased effects of ethanol on phase shifting following chronic drinking and sensitization of glutamate receptors following withdrawal (Lindsay and Prosser 2018).

Like alcohol, opioids exert many of their effects by impacting glutamatergic transmission. Modulation of glutamate signaling by opioids is mediated primarily through pre‐ or post‐synaptic binding of mu opioid receptors (MORs) to suppress neuronal activity (Chartoff and Connery 2014; Reeves et al. 2022). These MORs are highly expressed throughout the central nervous system and are well known to contribute to both alcohol and opioidergic rewarding effects (He and Whistler 2011; Wang et al. 2023; Xie, Wang, and Wang 2026). Of note, it has previously been shown that MOR activation inhibits ipRGCs (Berezin et al. 2022; Cleymaet et al. 2019, 2021), and that mice lacking MORs selectively in ipRGCs have altered sleep–wake activity (Berezin et al. 2022). Additionally, it has previously been shown that opioids, like alcohol, can inhibit phase shifting in the SCN (Meijer et al. 2000), further suggesting a critical role of these receptors in mediating circadian photoentrainment.

Here, we tested the hypothesis that alcohol and opioids alter SCN physiology by modulating both glutamatergic and opioidergic input. Using whole‐cell patch clamp electrophysiology and optogenetics in VGlut2‐Cre;Ai32 and MOR‐Cre;Ai32 mice, we examined how glutamatergic input and MOR^+^ fiber activation affect SCN firing following alcohol exposure conditions. We further evaluated the glutamatergic nature of these inputs and their sensitivity to pharmacological modulation by alcohol and the MOR agonists DAMGO and fentanyl. Our results reveal distinct, converging mechanisms through which depressants modulate glutamatergic signaling to the SCN, positioning the SCN as a novel node for substance‐induced circadian dysregulation.

## 2 Materials and Methods

### 2.1 Animals

VGlut2‐Cre, MOR‐Cre, and Ai32 mice were obtained from the Jackson Laboratory. Tail DNA samples were collected from mice, and PCR was conducted to determine the genotype. Mice were housed in same‐sex colonies under a 12 h:12 h light/dark schedule, with food and water available ad libitum. Mice were housed either in normal L/D (lights on at 06:00, off at 18:00) or reverse L/D (lights off at 11:00, lights on at 23:00), with times of recording normalized to Zeitgeber time (ZT), (lights on: ZT 0, lights off: ZT 12). All animal procedures in this study were approved by the Texas A&M University Institutional Animal Care and Use Committee. All procedures were conducted in agreement with the Guide for the Care and Use of Laboratory Animals, National Research Council, 1996.

### 2.2 Histology

Mice were anesthetized and perfused with 4% paraformaldehyde (PFA) in phosphate‐buffered saline (PBS). Brains were collected and submerged in 4% PFA/PBS solution overnight at 4°C before being transferred to 30% sucrose in PBS. After brains sunk, they were sliced on a cryostat into 50 μm sections. Confocal images were obtained with an Olympus Fluoview 1200 microscope using a 20× objective lens.

### 2.3 Intermittent Access Two‐Bottle Choice Drinking Procedure

To chronically expose mice to alcohol, an intermittent access to two‐bottle choice (IA2BC) drinking paradigm was utilized as previously described (Ben Hamida et al. 2013; Huang, Xie, Fernaine, Li, et al. 2026; Huang, Xie, Huang, Gangal, et al. 2026; Purvines et al. 2025; Xie et al. 2023). Following at least 6 weeks of group‐housed drinking, mice were individually housed to assess alcohol intake. Animals were given access to two bottles: one containing 20% alcohol in drinking water and the other containing drinking water only, provided three times a week (Monday, Wednesday, Friday) for 24 h. Control animals only received water. Both solutions were made fresh each week. To measure alcohol consumption, bottles were weighed before and after each session, and alcohol intake was calculated as the grams of alcohol consumed divided by the mouse's body weight in kilograms (g/kg/24 h). To account for drippage and evaporation, additional water and alcohol bottles were placed over empty cages during sessions, and bottles were weighed before and after each session to subtract the level of leakage from the amounts of liquid consumed.

### 2.4 Electrophysiology

Electrophysiological recordings were conducted as previously described (Essoh et al. 2025; Gangal et al. 2023; Huang et al. 2024; Huang, Xie, Huang, Chen, et al. 2026; Wang et al. 2011). Mice were deeply anesthetized with isoflurane before perfusion and brain extraction at least 1 h before neuron recordings (Figure S1A–E). Coronal SCN‐containing slices (250 μM) were then collected in ice‐cold cutting solution. Cutting solution contained (in mM): 40 NaCl, 148.5 sucrose, 4 KCl, 1.25 NaH_2_PO_4_, 25 NaHCO_3_, 0.5 CaCl_2_, 7 MgCl_2_, 10 glucose, 1 sodium ascorbate, 3 sodium pyruvate, and 3 myoinositol, saturated with 95% O_2_ and 5% CO_2_. Slices were incubated in a 1:1 mixture of cutting and external solution held at 32°C for 45 min before being transferred to pure external solution at room temperature for 15 min before use and the duration of the experiment. External solution consisted of (in mM): 125 NaCl, 4.5 KCl, 2.5 CaCl_2_, 1.3 MgCl_2_, 1.25 NaH_2_PO_4_, 25 NaHCO_3_, 15 sucrose, and 15 glucose, saturated with 95% O_2_ and 5% CO_2_.

In the recording chamber, slices were perfused with 32°C external solution at a flow rate of 1.5–2 mL/min. All recordings were conducted using a Multiclamp 700B amplifier controlled by pClamp 11.4 software (Molecular Devices). In cell‐attached recordings of SCN spontaneous firing, a K^+^ intracellular solution was used, consisting of (in mM): 123 potassium gluconate, 10 HEPES, 0.2 EGTA, 8 NaCl, 2 MgATP, 0.3 NaGTP (pH 7.3), with an osmolarity of 270–280 mOsm. Liquid junction potentials were not corrected for; all reported holding potentials reflect uncorrected command potentials. Recording configurations and solution compositions were kept constant across all experimental groups to ensure valid relative comparisons.

To measure synaptic transmission, whole‐cell patch clamp recordings were conducted in SCN neurons held at −50 mV (and recollected at −70 mV for verification purposes) in voltage‐clamp mode to measure AMPA‐mediated currents and +40 mV to measure NMDA currents (Xie et al. 2023). For paired‐pulse ratio experiments, light pulses were delivered at interstimulus intervals of 50, 100, 150, and 200 ms. A Cs‐based internal solution, containing (in mM): 119 CsMeSO_4_, 8 tetraethylammonium chloride, 15 HEPES, 0.6 EGTA, 0.3 Na_3_GTP, 4 MgATP, 5 QX‐314 chloride, and 7 phosphocreatine was used to measure excitatory input. To isolate excitatory input, 100 μM picrotoxin was present in the external solution to block the effects of GABA signaling. To measure optically induced synaptic transmission from VGlut2‐ and MOR‐positive input, blue light stimulation (473 nm, 5 ms pulse width) was delivered through the objective lens at varying intensities to stimulate Cre‐driven channelrhodopsin. The liquid junction potential was not corrected for. For experiments involving bath application of fentanyl (Figure 5), series resistance was monitored on every sweep using a brief hyperpolarizing voltage step (−5 mV, 200 ms) delivered 100 ms after sweep onset, ending ~700 ms before light stimulation so as not to interfere with the evoked response. Series resistance was calculated as *R*s = ΔV/ΔI, where ΔV is the −5 mV command step and ΔI is the peak current deflection it evoked. Rs was compared between the baseline (5 min) and the recording periods (5 min), and cells showing a > 20% change were excluded from analysis.

### 2.5 Statistical Analysis

All data were analyzed with consistent, predetermined parameters. Data from male and female subjects were combined for analysis. Treatments and groups were compared using either unpaired *t*‐tests, paired *t*‐tests, or two‐way ANOVA with repeated measures (two‐way RM ANOVA), followed by Tukey's *post hoc test*. Statistical analysis was conducted by SigmaPlot or SPSS. When data did not follow normal distribution, Generalized Linear Mixed Model (GLMM) tests were conducted to assess main effects and interactions, and Mann–Whitney Sum tests were used when data for unpaired *t*‐tests were not normally distributed. All data are expressed as the Mean ± SEM.

### 2.6 Manuscript Preparation

Throughout the preparation of this work, the authors used AI (ChatGPT and Google Gemini) to improve the clarity and readability of certain sections of the manuscript. After using this tool, the authors reviewed and edited the content as needed and took full responsibility for the content of the published article.

## 3 Results

### 3.1 Acute Alcohol Increases SCN Firing During the Daytime

Given the effects of acute alcohol drinking on sleep latency and other circadian processes (Koob and Colrain 2020; Meyrel et al. 2020), we first sought to determine the direct effect of acute alcohol on baseline SCN neuronal firing in slices. First, to determine the net effect of acute alcohol on the overall activity of the central clock, we measured the effects of bath‐applied alcohol on baseline spontaneous firing. We initially hypothesized that alcohol would lower SCN firing, as these neurons receive tonic glutamatergic input (Michel et al. 2002; Pennartz et al. 2001), and acute alcohol has been shown to inhibit the effects of glutamate (Prosser and Glass 2015). SCN firing can be largely divided into two phases, including the inactive locomotor stage during the day for rodents (characterized by high neural activity in the SCN, ZT 0–12), and the active locomotor stage during the night (characterized by low activity in the SCN (Belle et al. 2009; Colwell 2011; Van Oosterhout et al. 2012), ZT 12–0). Because of this, we recorded the effects of alcohol on acute firing during both phases.

Baseline action potential rate was measured via cell‐attached recording in slice, and after a steady baseline was observed, we applied 50 mM alcohol in ACSF to the bath, a dose corresponding with alcohol concentrations induced by binge drinking (Figure 1A). Surprisingly, SCN neurons recorded during inactive timepoints (ZT 7–12, daytime for mice, Figure S1A) showed a modest increase in raw and normalized firing frequency when exposed to alcohol (Figure 1B,D,E; BL vs. EtOH; *F*_(2,16)_ = 10.486, *p* < 0.05 (raw firing) and *F*_(2,16)_ = 10.623, *p* < 0.05 (normalized firing), 9 cells/7 animals), and this effect persisted during the wash period. In contrast, neurons recorded during the active phase (ZT 12–20) did not have a significant increase in firing (Figure 1C,D,F; BL vs. EtOH, *F*_(2,22)_ = 0.883, *p* > 0.05 (raw firing) and *F*_(2,22)_ = 0.688, *p* > 0.05 (normalized firing) by one‐way RM ANOVA, 12 cells/7 animals). When active and inactive timepoints were analyzed together to determine interactions, there was only a trend in the effect of recording time (Figure S2A, *F*_(2,38)_ = 3.03, *p* = 0.06 (normalized firing), *F*_(2,38)_ = 2.88, *p* = 0.07 (raw firing)). This was mostly due to differences in firing during the washout period, as post hoc tests found no difference in normalized or raw SCN firing frequency during alcohol application. Interestingly, we noted a sex difference in response to alcohol during the active stage, with females showing increased spontaneous firing compared to males during alcohol bath application (Figure S2B, *t*_(9)_ = −2.349, *p* < 0.05). This effect did not persist in the inactive stage, with males and females showing similar increases in firing in response to alcohol (Figure S2C, *t*_(5)_ = 0.106, *p* = 0.92).

**FIGURE 1:**
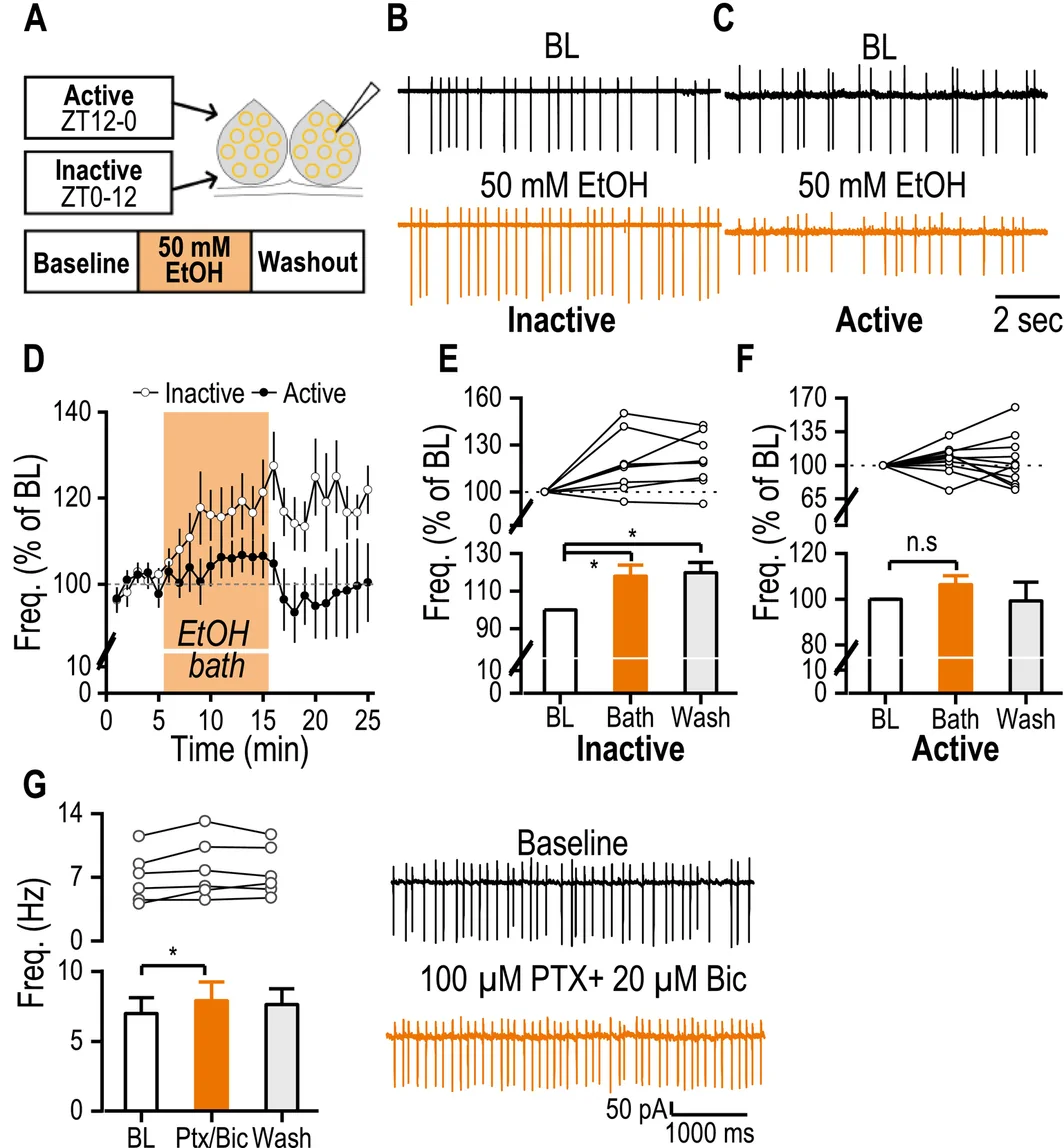
Alcohol acutely increases the spontaneous firing of SCN neurons during the inactive circadian stage. (A) Schematic of experimental design. SCN slices were recorded under active (ZT12–ZT0) and inactive (ZT0–ZT12) circadian phases. Slices were recorded during baseline conditions, during application of 50 mM EtOH, and during EtOH washout. (B) Representative traces of SCN spontaneous firing under baseline conditions (BL) and during bath application of 50 mM ethanol (EtOH) in the inactive stage. Neurons recorded during the inactive phase showed increased firing during EtOH administration compared to baseline. (C) Representative traces of SCN spontaneous firing under baseline conditions and during bath application of 50 mM ethanol in the active stage. Neurons recorded during the active phase showed no change in firing during EtOH administration compared to baseline. (D) Time course of normalized spontaneous firing rate before, during, and after application of 50 mM EtOH. Inactive neurons increased their firing during EtOH administration, whereas active neurons did not; *n =* 9/7 (inactive, 2 male and 4 female, 1 unknown) and 12/7 (active, 4 male and 2 female, 1 unknown). (E) Bar graph comparing the average normalized spontaneous firing rate at baseline (BL), during ethanol application (EtOH), and after washout for neurons in the inactive stage. Inactive neurons significantly increased firing during EtOH administration and washout periods (*p < 0.05 by one‐way RM ANOVA, *n =* 9/7). (F) Bar graph comparing the average normalized spontaneous firing rate at baseline (BL), during ethanol application (EtOH), and after washout for neurons in the active stage. Active neurons had no significant change in firing during EtOH administration and washout periods (^n.s^ *p* > 0.05 by one‐way RM ANOVA, *n =* 12/7). (G) Left, bar graph comparing the average raw spontaneous firing rate at baseline (BL), during picrotoxin (PTX) + bicuculline (Bic) application, and after washout. PTX and Bic significantly increased firing rates of SCN neurons (*p < 0.05 by paired *t*‐test, *n* = 6 cells). Right, representative traces of SCN spontaneous firing before (baseline) and during application of the GABA_A receptor antagonists PTX (100 μM) and BIC (20 μM).

We next examined whether these effects on SCN firing frequency were due to enhanced GABA release induced by alcohol. Alcohol acutely facilitates GABAergic signaling (Kelm et al. 2011), and GABA has been reported to have excitatory actions on some SCN neurons (Wagner et al. 1997). However, when we bath applied GABA antagonists picrotoxin (PTX) and bicuculline (BIC), this treatment appeared to increase SCN firing (Figure 1G). There was a significant increase in firing frequency from baseline measurements during PTX and BIC application (Figure 1G, *t*_(5)_ = −2.785, *p* < 0.05). However, the effect was not significant when one‐way RM ANOVA analysis was conducted to include washout measurements, possibly due to insufficient sample size (effect of treatment, *F*_(2,10)_ = 3.440, *p* > 0.05). This suggests that GABA may be inhibitory in the SCN, which has also been previously demonstrated (Ono et al. 2018). Thus, it is unlikely that alcohol increased SCN firing through enhanced GABA signaling. Overall, these results suggest that alcohol has an excitatory effect on the master circadian clock during inactive times of day, and that this effect is unlikely to be due to altered GABA release.

### 3.2 Optogenetic Activation of Retinal Glutamatergic Inputs Excites SCN Neurons

A consistent aspect of SCN physiology is its glutamatergic input from the RHT (Fernandez et al. 2016; Pu and Pickard 1996; Tetsuo Shirakawa 1994). Given the importance of this pathway for entrainment to the solar cycle, we next sought to examine the effects of chronic drinking on this pathway. To study chronic effects of alcohol on glutamatergic input to the SCN, we first confirmed the utility of a VGlut2‐Cre;Ai32 optogenetic mouse line (Lemieux and Bretzner 2019) in analyzing this pathway. VGlut2 is a gene encoding a vesicular glutamate transporter and is known to be expressed in RHT ipRGCs (Engelund et al. 2010), so Ai32 allowed us to optogenetically stimulate RHT ipRGCs (Figure 2A). Neurons were recorded from ZT 6–12 (Figure S1B). Single 5‐ms blue light pulses (470 nm) reliably evoked excitatory postsynaptic currents (EPSCs) in SCN neurons, which were abolished by the AMPA receptor antagonist DNQX, confirming glutamatergic transmission (Figure 2B). Burst stimulation (10 Hz, 30 s) produced a robust, reversible increase in spontaneous action potential (sAP) firing during light delivery (Figure 2C), resulting in a significant rise in sAP frequency that returned to baseline after light stimulation (Figure 2D–E; *F*_(2,16)_ = 19.12, *p* < 0.001, 9 cells/3 animals). These findings demonstrate that optogenetic stimulation of endogenous glutamatergic RHT afferents reliably and reversibly evokes excitatory responses in SCN neurons.

**FIGURE 2:**
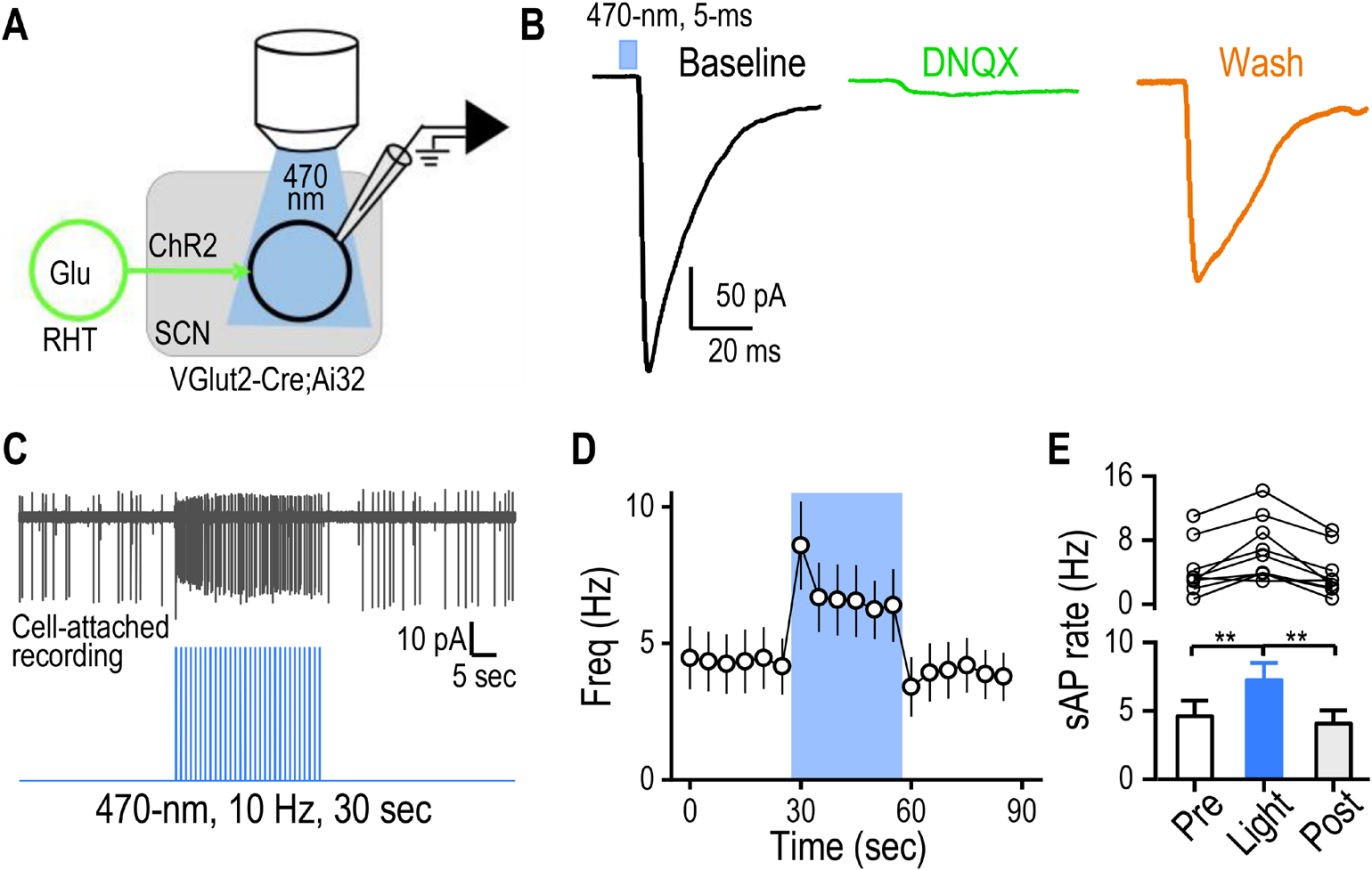
Optogenetic stimulation of retinohypothalamic tract (RHT) glutamatergic inputs excites SCN neurons in VGlut2‐Cre;Ai32 mice. (A) Schematic of the experimental design for optogenetic stimulation of the RHT and recording from SCN neurons. SCN slices from ~12‐month‐old VGlut2‐Cre;Ai32 mice of both sexes were recorded in response to 470‐nm light stimulation of RHT inputs. (B) Optogenetic stimulation of RHT inputs induced EPSCs in SCN neurons and this response could be blocked by the AMPAR antagonist DNQX (20 μM). (C) Representative trace of spontaneous firing in SCN neurons before, during, and after burst stimulation of RHT input. (D) Time course of spontaneous firing rate in SCN neurons before, during, and after burst stimulation of RHT input. *n* = 9/3. (E) Summary data showing that burst stimulation of the RHT significantly and reversibly increases the spontaneous firing rate of SCN neurons. **p < 0.01 by post hoc test following one‐way RM ANOVA.

### 3.3 Chronic Alcohol Exposure Reduces Glutamatergic Synaptic Strength in the SCN

Given the critical role of glutamatergic signaling for SCN entrainment, we next investigated whether chronic alcohol exposure alters VGlut2+ input to the SCN. Mice were given > 8 weeks of 20% alcohol using the intermittent access two‐bottle choice (IA2BC) drinking procedure, which models human patterns of drinking (Vierkant et al. 2023), or water alone as a control (Figure 3A). In this paradigm, mice are given 24 h access to either 20% alcohol or water followed by 24–48 h of withdrawal. For each session, alcohol was provided between ZT 11.3 and ZT 16 and removed 24 h later. Alcohol‐exposed mice reached high levels of alcohol consumption and preference prior to recordings (Figure 3B,C), showing increased preference for alcohol over water during alcohol access periods (*t*_(9)_ = −4.229, *p* < 0.05). Recordings from alcohol‐exposed mice were performed 48 h after alcohol was removed to assess withdrawal effects. All experiments were conducted between ZT 13–15, the early active phase, when SCN neurons are most responsive to photic and glutamatergic input (Figure S1C). Whole‐cell voltage‐clamp recordings were obtained from neurons in the SCN core and held at −50 mV. To isolate excitatory synaptic transmission, the GABA_A_ receptor antagonist picrotoxin (0.1 mM) was present during the entire recording.

**FIGURE 3:**
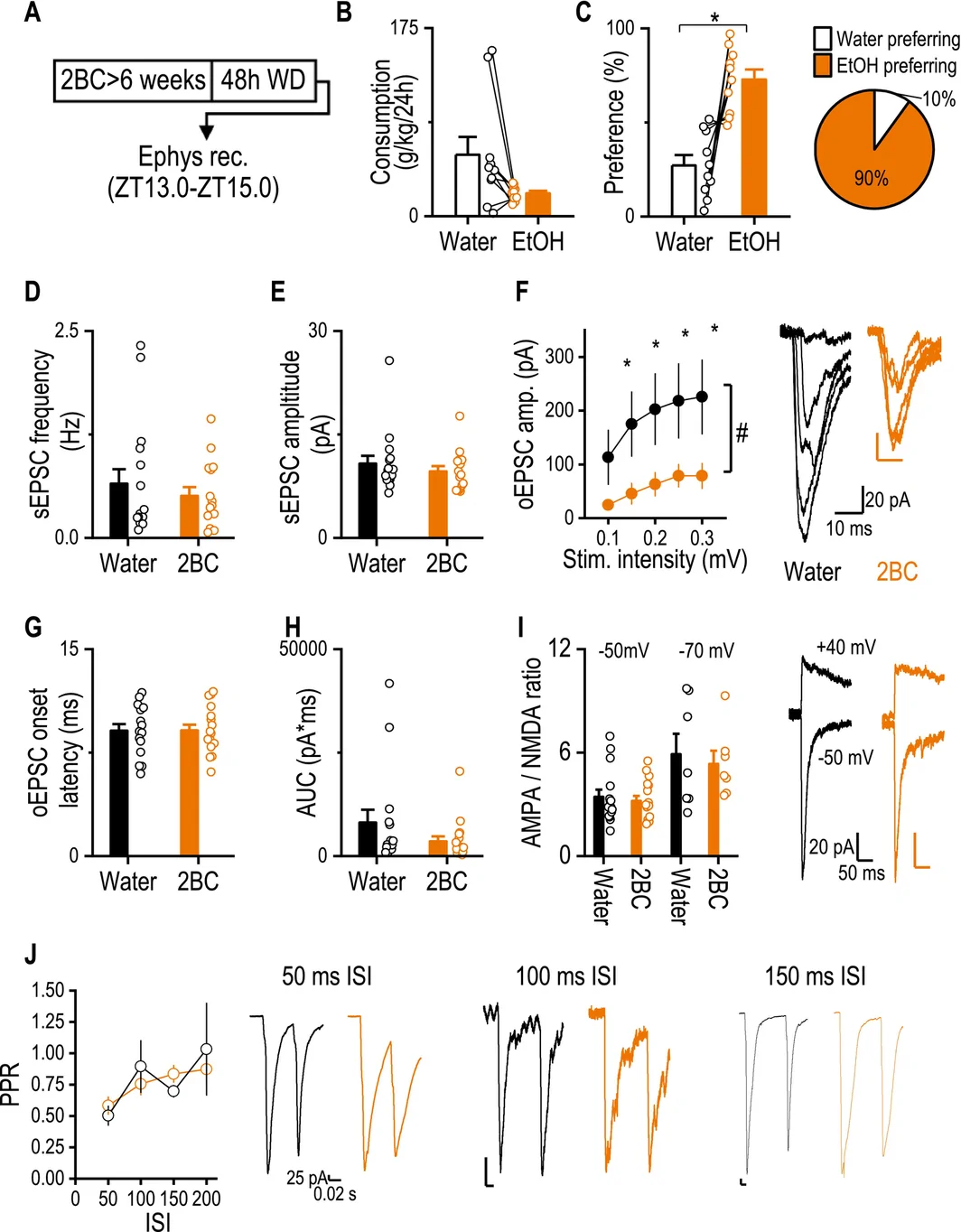
Chronic alcohol exposure reduces glutamatergic synaptic strength in the SCN. (A) Schematic of the experimental timeline. VGlut2‐Cre;Ai32 mice were exposed to intermittent access to two‐bottle choice (2BC) of alcohol or water (water alone for controls) for > 6 weeks. Slices were collected to assess excitatory input to SCN neurons following 48 h of withdrawal. (B) Intermittent access led to physiologically relevant levels of EtOH consumption. (C) Intermittent access to 2BC led to EtOH preference over water in the vast majority of animals. *p < 0.05 by paired t‐test, *n* = 10. (D) 2BC did not alter sEPSC frequency compared to water‐only controls (not significant by Mann–Whitney Sum Test). *n* = 16/4 (water, 2 male, 2 female) 15/4 (2BC, 3 male, 1 female). (E) 2BC did not alter sEPSC amplitude compared to water‐only controls (not significant by Mann–Whitney Sum Test). *n* = 16/4 (water, 2 male, 2 female) 15/4 (2BC, 3 male, 1 female). (F) Left, Input–output AMPAR‐mediated oEPSCs were reduced in SCN neurons of 2BC mice. *p/^#^ *p* < 0.05 by two‐way RM ANOVA and GLMM, *n* = 15/4 for water, 2 male, 2 female, and 17/4 for 2BC, 3 male, 1 female. Right, sample trace of neurons obtained from water and 2BC mice showing reduced oEPSC amplitude. (G) did not alter oEPSC onset latency compared to water‐only controls (not significant by *t*‐test). *n* = 15/4 (water, 2 male, 2 female) 17/4 (2BC, 3 male, 1 female). (H) 2BC did not alter oEPSC area under the curve (AUC) compared to water‐only controls (not significant by Mann–Whitney Sum Test). *n* = 15/4 (water, 2 male, 2 female) 17/4 (2BCb, 3 male, 1 female). (I) Left, AMPA/NMDA ratio was not affected by 2BC (*p* > 0.05 by Mann–Whitney Sum Test), *n* = 15/4 for water (2 male and 2 female) and 16/4 for 2BCbc (3 male and 1 female) in −50 mV group, 7/2 for water (2 male and 0 female) and 7/2 for 2BC (1 male and 1 female) in −70 mV group. Right, sample traces from representative water and 2BCbc‐exposed neurons. (J) Left, averaged data showing that 2BCb did not alter the paired pulse ratio at intersweep intervals of 50, 100, 150, or 200 ms (*p* > 0.05 by two‐way RM ANOVA), *n* = 16/7 for water (3 male and 4 female) and 18/6 for 2BC (5 male and 1 female). Right, sample traces showing no differences in PPR between water and 2BC‐exposed neurons.

Prior to manipulating RHT input, we first measured spontaneous excitatory synaptic input to SCN neurons in both alcohol withdrawal and water control groups. Analysis of spontaneous EPSCs (sEPSCs) revealed no significant group differences in frequency (Figure 3D; Mann–Whitney *U* = 118.00, *n*_1_ = 16, *n*_2_ = 15, *p* = 0.953, 31 cells/8 animals) or amplitude (Figure 3E; Mann–Whitney *U* = 101.00, *n*_1_ = 15, *n*_2_ = 16, *p* = 0.465). We then stimulated VGlut2+ input with escalating intensities of blue light pulses to assess excitatory signaling in water and alcohol groups. Compared to controls, neurons from alcohol‐exposed mice displayed significantly lower excitatory responses (Figure 3F; *p* = 0.026 by GLMM). This reduction was not accompanied by altered onset latency (Figure 3G, *t*_(30)_ = −0.0487, *p* = 0.962) or changes in AUC (Figure 3H, Mann–Whitney *U* = 89.00, *n*_1_ = 15, *n*_2_ = 17, *p* = 0.151), although AUC seemed to show a slight trend in reduction. This reduction did not appear to result from selective postsynaptic AMPAR changes, as AMPA/NMDA ratios were comparable across groups (Figure 3I; Mann–Whitney *U* = 111.00, *n*_1_ = 15, *n*_2_ = 16, *p* = 0.737; *t*_(12)_ = 0.401, *p* = 0.696). In addition, paired‐pulse ratios did not differ between groups at intersweep intervals of 50, 100, 150, or 200 ms (Figure 3J; *F*_(3,74)_ = 1.143, *p* = 0.337), suggesting that presynaptic release probability was not altered. Together, these findings indicate that chronic alcohol drinking reduces optogenetically evoked excitatory responses following stimulation of VGlut2‐expressing afferents to the SCN.

### 3.4 MOR+ Projections to the SCN Bidirectionally Modulate Neuronal Firing

Opioids, like alcohol, are central nervous system depressants and can produce long‐term disruptions in sleep when abused (Eacret et al. 2020). Additionally, the endogenous opioid system is repeatedly implicated with alcohol use disorder (Gianoulakis 2009), with one of the few FDA‐approved pharmacological treatments for AUD, naltrexone, being an MOR antagonist. In vivo alcohol exposure has been shown to diminish MOR‐mediated suppression of excitatory synaptic transmission in various corticostriatal synapses (Muñoz et al. 2020), and chronic drinking impairs MOR coupling with G‐proteins in multiple brain areas (Saland et al. 2008). This sensitivity of MORs to alcohol may have important implications for circadian entrainment, as it is known that MOR activation can inhibit phase shifting in the SCN (Berezin et al. 2022; Meijer et al. 2000), with fentanyl injections mid‐day inducing phase advances in wheel‐running activity rhythms. However, it is unknown whether opioidergic signaling alters SCN physiology and if this modulatory influence is sensitive to alcohol. To address this gap in knowledge, we next sought to determine how MOR+ neuronal populations influence the SCN.

Fixed‐tissue histology in MOR‐Cre;Ai14 mice was used to determine the expression of the MOR receptor and fibers in the SCN. We observed dense MOR+ fibers within the RHT and a small subset of MOR+ neurons within the SCN (Figure 4A). To validate that presynaptic MOR inputs project to the SCN and test how they affect neuronal activity, we used MOR‐Cre;Ai32 mice to optogenetically stimulate MOR+ fibers while recording spontaneous firing of SCN neurons (Figure 4B). Neurons were recorded between ZT 6 and ZT 15 (Figure S1D). Blue light burst stimulation produced heterogeneous effects on firing, where 46.2% of neurons increased firing by > 10%, 23.1% decreased firing by > 10%, and 30.8% showed no significant change (Figure 4C,D, 13 cells/7 animals). These effects were consistent across repeated stimulation sweeps and quickly recovered during the post‐stimulation period (Figure 4E–H).

**FIGURE 4:**
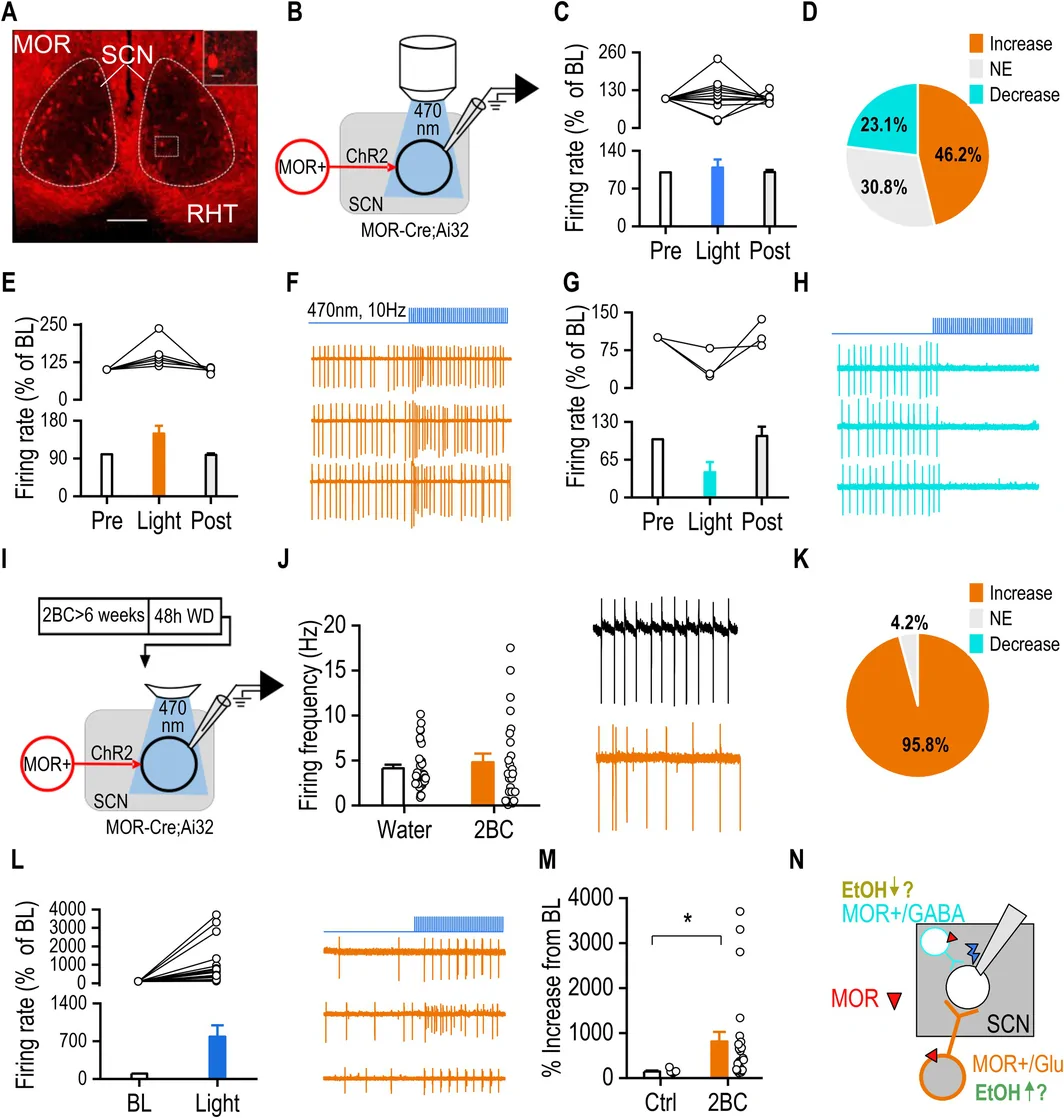
Activation of MOR input to the SCN produces bidirectional effects on SCN neuronal firing. (A) Sample image of MOR+ fibers and neurons in the SCN and surrounding area. Image taken from an MOR‐Cre;Ai14 mouse, inset scale bar: 10 μm. (B) Schematic depicting experimental design. SCN slices were prepared from 7‐ to 8‐month‐old MOR‐Cre;Ai32 mice of both sexes. (C) Light stimulation of MOR input has no significant effect on firing rate when all neurons were combined together (*p* > 0.05 by Friedman Repeated Measures Analysis of Variance on Ranks, *n* = 13/7). (D) Pie chart showing differential responses of SCN neurons to light stimulation. Out of all neurons recorded, the majority showed an increase of greater than 10% during light stimulation (*n* = 13/7). (E) Light stimulation induced a significant increase in firing in a subset of SCN neurons, *n* = 6/3. (F) Sample traces of three sweeps in an SCN neuron showing repeated, reversible excitation when MOR input was optogenetically activated. (G) A smaller subset of SCN neurons was reversibly inhibited by light stimulation, *n* = 3/3. (H) Sample traces of three sweeps in an SCN neuron showing repeated, reversible inhibition when MOR input was optogenetically activated. (I) Schematic depicting experimental design. SCN slices were prepared from MOR‐Cre;Ai32 mice of both sexes following > 6 weeks of intermittent access to two‐bottle choice (2BC) alcohol drinking. (J) There was no significant difference in SCN spontaneous firing in water and 2BC groups (not significant by Mann–Whitney sum test). *n* = 37/7 for water, 25/4 for 2BC. (K) The majority of SCN neurons in the 2BC group showed increased spontaneous firing following MOR+ stimulation. (L) Light stimulation induced a significant increase in the majority of SCN neurons in the 2BC group. Compared to water‐drinking animals, SCN neurons that showed increased firing following MOR+ stimulation showed higher increases from baseline in 2BC animals. *p < 0.05 by Mann–Whitney sum test. *n* = 6/3 for water, 23/4 for 2BC. (M) Proposed neurocircuit of converging glutamatergic and GABAergic MOR+ input to the SCN.

To test if chronic alcohol consumption can modulate SCN response to presynaptic MOR stimulation, we put MOR‐Cre;Ai32 animals through intermittent‐access 2BC. When these animals had been in 2BC for > 6 weeks, we similarly optogenetically stimulated MOR+ fibers while recording spontaneous firing of SCN neurons (Figure 4I). Water‐drinking and alcohol‐drinking animal SCN neurons showed similar baseline spontaneous firing (Figure 4J, Mann–Whitney *U* = 433.00, *n*1 = 37, *n*2 = 25, *p* > 0.05). However, compared to the water‐drinking animals, over 90% of SCN neurons in the alcohol‐drinking group showed increased spontaneous firing rates following MOR stimulation (Figure 4K,L, 24 cells/4 animals). Interestingly, this increase in firing rate was significantly larger in the alcohol‐drinking group compared to water‐drinking increases (Figure 4M, Mann–Whitney *U* = 15.00, *n*1 = 6, *n*2 = 23, *p* < 0.05).

The MOR‐Cre;Ai32 mouse line we used stimulates all MOR+ neurons in the coronal brain slice rather than neurons in one afferent region. Altogether, however, these findings suggest that excitatory effects potentially arise from MOR^+^ RHT afferents, whereas it is possible that inhibitory effects arise from MOR+ GABAergic afferents from other regions (Eacret et al. 2023). Similarly, it is possible that chronic alcohol drinking upregulates glutamatergic inputs or downregulates GABAergic inputs (Figure 4N), leading to increased SCN neuronal activity following MOR stimulation.

### 3.5 MOR Agonists Suppress Glutamatergic Input to the SCN

Because we observed that a plurality of SCN neurons had excitatory responses to MOR+ input and given our previous findings on altered glutamatergic and opioidergic input following chronic alcohol, we next characterized the excitatory component of MOR+ input to the master clock (Figure 5A). Neurons were recorded between ZT 14 and ZT 18. Optogenetic activation of MOR+ terminals elicited robust oEPSCs (Figure 5B) that were diminished by the AMPAR antagonist NBQX (Figure 5C,D), confirming a glutamatergic component to excitatory MOR+ input.

**FIGURE 5:**
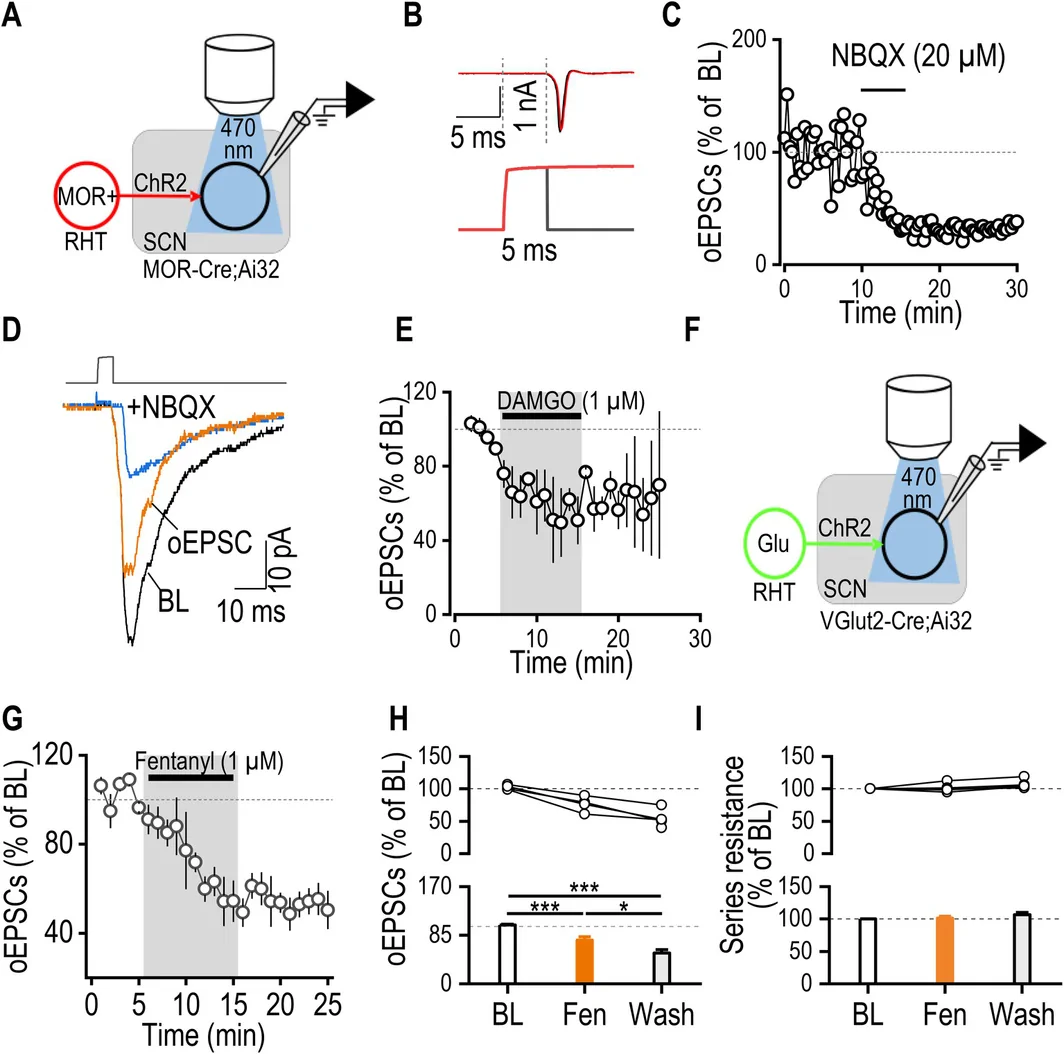
MOR+ inputs form glutamatergic synapses on SCN neurons that can be suppressed by MOR agonists. (A) Schematic depicting experimental design. SCN slices were prepared from an MOR‐Cre;Ai32 mouse. (B) Sample trace of an optically induced EPSC (oEPSC) in an SCN neuron, showing ~5 ms latency. *n* = 1/1. (C) Bath application of AMPAR antagonist NBQX (20 μM) reduced oEPSC amplitude in an SCN neuron. *n* = 1/1. (D) Sample trace of a recorded neuron showing the baseline (BL), the response during NBQX application, and the subtraction of the baseline and NBQX response to equal the actual oEPSC response. *n* = 1/1. (E) The MOR agonist DAMGO reduced the amplitude of oEPSCs following MOR stimulation. *n* = 2/1. (F) Schematic depicting experimental design. SCN slices were prepared from VGlut2‐Cre;Ai32 mice. (G) Time course showing bath application of fentanyl (1 μM) acutely and persistently suppresses glutamatergic input to SCN neurons. (H) Fentanyl bath application significantly reduced the amplitude of oEPSCs compared to baseline (BL). This effect persisted throughout the washout (wash) period (***p < 0.001 by one‐way RM ANOVA, *n* = 5/3). (I) Series resistance was calculated during baseline (BL), fentanyl application (Fen), and washout (Wash), and neurons showing > 20% change from baseline series resistance were excluded from analysis (*n* = 6/4).

Next, we wanted to examine the effects of MOR+ agonists on excitatory input to the SCN. Bath application of the MOR agonist DAMGO (1 μM) reduced oEPSC amplitudes (Figure 5E), suggesting glutamatergic input to the central clock expresses functional MORs. Last, we tested the effect of the synthetic opioid fentanyl on glutamatergic input to SCN neurons. SCN slices were prepared from 3‐month‐old VGlut2‐Cre;Ai32 mice (Figure 5F) and recordings were conducted as described in Figure 3. After establishing a stable baseline for ~5 min, fentanyl (1 μM) was bath‐applied, producing a gradual reduction in oEPSC amplitude that reached a plateau during the final 4 min of the 10‐min application (Figure 5G). This suppression continued during the subsequent washout period, with no recovery toward baseline (Figure 5H, *F*_(2,8)_ = 57.046, *p* < 0.001). Importantly, series resistance was calculated during baseline, fentanyl application, and washout periods to ensure results were not due to deterioration of recording quality (Figure 5I), and neurons showing > 20% change in series resistance from baseline were excluded from further analysis. Only one neuron reached this exclusion criteria and was removed from analysis. Together, these results indicate that acute fentanyl exposure produces a robust and persistent suppression of glutamatergic input to SCN neurons, although the available washout recordings do not establish pharmacological reversibility of this effect.

## 4 Discussion

This work studied acute and chronic effects of alcohol on SCN physiology and characterized endogenous input to the master clock using optogenetics. Additionally, our findings reveal that central nervous system depressants modulate SCN activity by disrupting excitatory glutamatergic input. These findings highlight mechanisms through which alcohol and opioids may alter circadian timing, potentially leading to the known sleep disturbances that occur with SUDs. Notably, however, although both substance classes target circuits that influence SCN excitability, they seem to do so via distinct but converging pathways.

We first demonstrated that acute ethanol exposure enhances SCN firing in a manner unlikely to reflect enhanced GABAergic signaling, suggesting a facilitation of intrinsic excitability or excitatory input. This is particularly notable given that GABA is the predominant neurotransmitter in the SCN (Ono et al. 2018). Interestingly, however, this effect only occurred during the inactive circadian phase, indicating that acute alcohol may most significantly impair circadian function during times of rest. Considering that SCN neurons show increased firing during the inactive stage (Colwell 2011), this could contribute to acute sleep disturbances seen with alcohol use, such as short sleep duration (He et al. 2019). Interestingly, however, this excitation is unlikely to reflect an acute shift in the phase of the SCN, as such phase adjustments occur over hours, far longer than the recording timescale. Rather, alcohol may exert a lasting modulatory effect on one or more of the ion channels or metabotropic receptors that govern SCN excitability. Furthermore, we cannot exclude the possibility that the increase in spontaneous firing observed during acute alcohol application involves modulation of inputs to the SCN. While further work is needed to understand the mechanism of this phenomenon, it is possible that this effect could, in part, mediate the effects of acute alcohol intoxication on circadian‐related behaviors.

Interestingly, we also found that chronic alcohol exposure had minimal effects on SCN spontaneous firing rates or amplitude when neurons were recorded during the active stage. However, by utilizing VGlut2‐Cre;Ai32 mice to selectively stimulate VGlut2‐expressing afferents to the SCN, we found that chronic alcohol drinking reduces AMPAR‐mediated optogenetically evoked excitatory responses. These findings are particularly interesting as they indicate that long‐term alcohol use may potentially modulate photic entrainment. While the reduction we observed was AMPA‐dependent and NMDARs are typically regarded as the primary mediator of photic shifts, AMPAR activation also contributes to photic resetting. Moreover, blockade of AMPARs has been shown to attenuate light responses (Colwell and Menaker 1992) and exogenous AMPAR activation can induce phase shifts in the SCN (Mizoro et al. 2010). Because TTX/4‐AP isolation was not performed, however, polysynaptic contributions to these responses cannot be excluded. Thus, these findings are best interpreted as altered excitatory responses recruited by VGlut2+ afferent stimulation. However, these findings provide a potential synaptic basis for circadian dysregulation, showing that chronic alcohol weakens excitatory drive onto SCN neurons, compromising a pathway essential for entrainment of brain and body rhythms to the external environment.

Opioidergic modulation of SCN function has been less well characterized in the previous literature despite common reports of sleep disturbances that accompany opioid use (Javaheri and Cao 2022). Here, we confirmed the presence of MOR+ fibers in the SCN, including in the RHT pathway (Berezin et al. 2022; Bergum et al. 2022), suggesting a direct interaction between opioid‐sensitive circuits and photic entrainment. Further, we showed that MOR+ inputs to the SCN can evoke both excitatory and inhibitory responses, suggesting circuit heterogeneity. Although nearly 50% of recorded neurons showed increased firing following MOR+ fiber stimulation, around 23% demonstrated decreased firing. Given the high expression of MOR fibers in the RHT, the observed excitatory response is likely mediated by ipRGCs (Bergum et al. 2022), whereas inhibitory responses may come from the sparse MOR+ GABAergic SCN neurons observed in our imaging, polysynaptic recruitment of local SCN neurons by the RHT, or other upstream GABAergic MOR+ input (Hanna et al. 2017; Pu and Pickard 1996). It is also possible that the observed excitatory response arises from other glutamatergic upstream neurons outside of the RHT, such as the anterior paraventricular thalamus (Moga et al. 1995). Together, however, these findings indicate that MOR^+^ inputs provide both excitatory and inhibitory modulation of SCN firing, with glutamatergic transmission subject to suppression by MOR activation. In the context of AUD, where alcohol interacts with the endogenous opioid system and disrupts circadian rhythms, these results suggest a potential mechanism by which opioid‐sensitive inputs contribute to alcohol's modulation of the central circadian clock. Interestingly, we found that chronic alcohol drinking seemed to alter SCN response to these opioid‐sensitive inputs, with more neurons having increased excitatory response to MOR+ stimulation. This further suggests an interaction between the endogenous opioid system and circadian system disruption in AUD.

Importantly, we also found that MOR+ terminals form functional glutamatergic synapses that are sensitive to suppression by MOR activation. Both the MOR agonist DAMGO and the synthetic opioid fentanyl suppressed oEPSC amplitude from MOR+ and VGlut2+ input, respectively. This suppression is consistent with known mechanisms of presynaptic inhibition in other brain regions (Boxwell et al. 2013), further verifying a role for opioidergic signaling in modulating SCN function.

Together, these data support a model in which depressants alter SCN function by targeting excitatory glutamatergic input. These results lay important groundwork for how alcohol and opioids may affect the SCN. We showed that alcohol produces both acute excitation and chronic suppression depending on the length of alcohol exposure. Additionally, we found that MOR+ inputs bidirectionally regulated SCN excitability, and that this regulation may be altered by chronic alcohol consumption. Further, we showed that MOR agonists can suppress glutamatergic input to the SCN. This modulation may underlie the well‐documented circadian disturbances observed in SUDs and may contribute to altered sleep, hormone regulation, and behavioral rhythms in affected individuals.

Limitations and future directions include the need to map specific MOR+ input sources, determine the molecular basis for the divergent excitatory/inhibitory responses to MOR+ stimulation, and test whether these physiological alterations produce measurable changes in circadian outputs. Additionally, although both fentanyl and DAMGO suppressed glutamatergic input to the SCN, the available washout recordings do not establish pharmacological reversibility, and the absence of antagonist pretreatment prevents us from definitively establishing MOR specificity. Future studies incorporating opioid receptor antagonist pretreatment will be important to directly address this question. It will also be important to explore whether these effects are similarly generalized to other depressant drugs (e.g., benzodiazepines), thus extending the implications of this work to broader clinical relevance. Furthermore, we cannot exclude the possibility that the increase in spontaneous firing observed during acute alcohol application involves modulation of inputs to the SCN rather than a direct effect on SCN neurons. Finally, a key factor to consider when interpreting our results include that SCN neurons are a heterogeneous population, meaning it is possible that neurons recorded at each timepoint are not representative of average activity across the nucleus. Along this line, although we uncovered an apparent sex difference in SCN spontaneous firing during alcohol bath application, our study was not designed to examine sex differences and thus lacks the necessary power for additional sex‐related analyses. Overall, however, our study identifies the SCN as a previously underappreciated target of alcohol and opioid modulation and reveals glutamatergic transmission as a shared mechanistic substrate through which these depressants impair circadian function.

## Funding

This work was supported by NIAAA R01AA021505, U01AA025932, R01AA032498, R01AA030293 and R01DA064912.

## Conflicts of Interest

The authors declare no conflicts of interest.

## Supporting information


**Figure S1:** ZT times for electrophysiology recordings. (A) Scatter graph depicting the ZT time at which the first neuron for each animal was recorded in Figure 1. In Figure 1, neurons were recorded in either the active stage (lights off, ZT 12.00–0.00) or the inactive stage (lights on, ZT 0.00–12.00). Times at which the lights were on is depicted in yellow shading. (B) Scatter graph depicting the ZT time at which each neuron was recorded in Figure 2. In Figure 2, neurons were recorded in the late inactive stage. (C) Scatter graph depicting the ZT time at which the first neuron for each animal was recorded in Figure 3. In Figure 3, neurons were recorded in the early active stage. (D) Scatter graph depicting the ZT time at which each neuron was recorded in Figure 4. In Figure 4, the majority of neurons were recorded in the late inactive stage, with two being recorded at the beginning of the active stage. (E) Scatter graph depicting the ZT time at which each neuron was recorded in Figure 5. In Figure 5, neurons were recorded in the active stage.
**Figure S2:** SCN neurons in the active stage show increased firing rates in female mice compared to male mice. (A) Time course of raw spontaneous firing rate before, during, and after application of 50 mM EtOH. When not split into active or inactive stages, there were no significant effects on firing. (B) During the active stage, SCN neurons from female mice showed increased firing during EtOH bath application compared to male mice. (C) During the inactive stage, SCN neurons showed no sex differences in firing rate during EtOH bath application.

## Data Availability

The data that support the findings of this study are available from the corresponding author upon reasonable request.
