# Supplementary material for: Alcohol and Opioids Modulate Excitatory Inputs to the SCN
Source: Alcohol Clin Exp Res (Hoboken). 2026 Nov 10;50(11):e70447. doi: 10.1111/acer.70447 (PMC13751877; doi:10.1111/acer.70447)
Supplement: Supplementary file 1 [file ACER-50-0-s001.pdf]

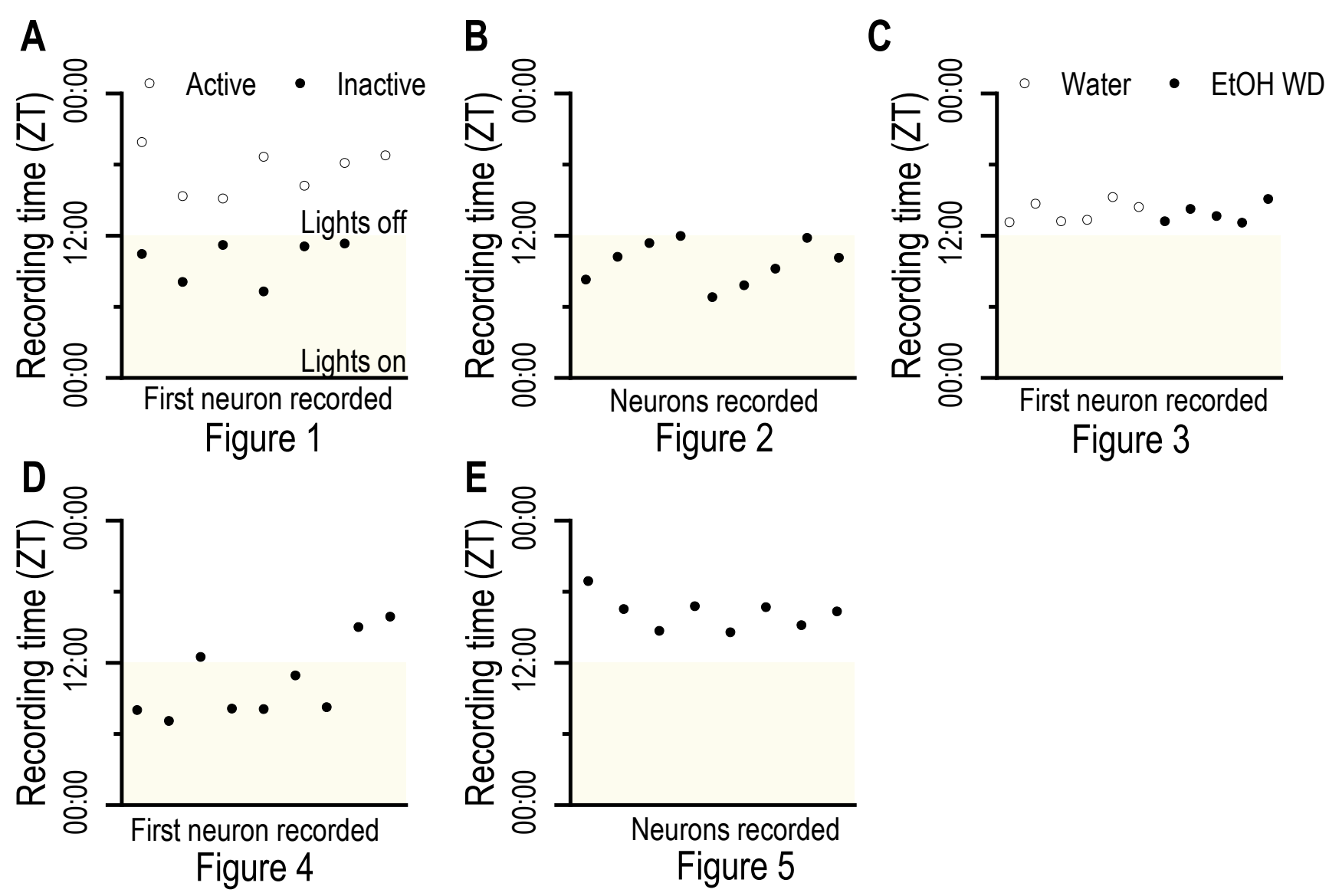

A
B
C
D
E
Active
Inactive
Water
EtOH WD
Recording time (ZT)
00:00
12:00
Lights off
Lights on
First neuron recorded
Neurons recorded
Figure 1
Figure 2
Figure 3
Figure 4
Figure 5

Figure S1

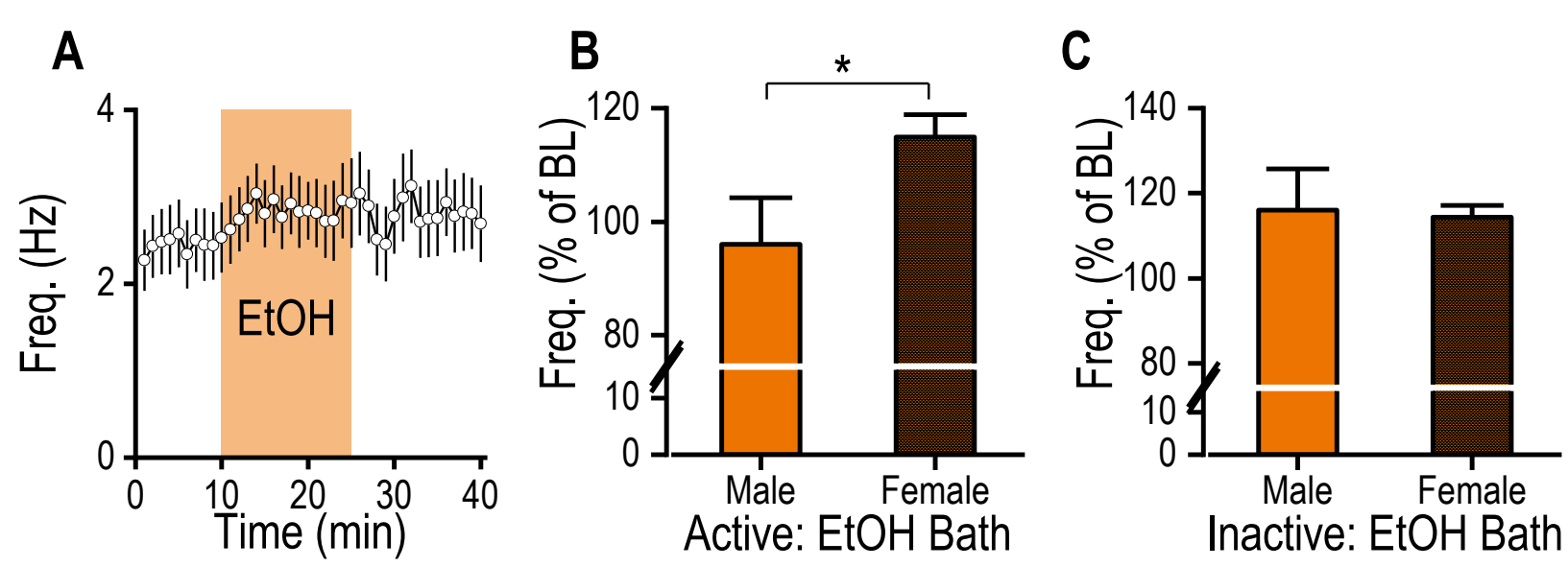
A
Freq. (Hz)
4
2
0
EtOH
0
10
20
30
40
Time (min)
B
*
Freq. (% of BL)
120
100
80
10
0
Male
Female
Active: EtOH Bath
C
Freq. (% of BL)
140
120
100
80
10
0
Male
Female
Inactive: EtOH Bath

Figure S2
